# Use of Small Organic Molecules to Improve Pancreatic Beta Cell Resilience to IAPP-Induced Proteotoxic Stress

**DOI:** 10.3390/ijms27073004

**Published:** 2026-03-26

**Authors:** Kai Valshon, Kaili Kondrot, Hana Uehara, Michael Alexander, Hirohito Ichii

**Affiliations:** 1 Division of Endocrinology, Rady Children’s Health Orange County, Orange, CA 92868, USA; kaiser.valshon@choc.org; 2 Pediatric Endocrinology Fellowship Program, University of California Irvine, Orange, CA 92868, USA; 3 Department of Surgery, University of California Irvine, Orange, CA 92868, USA; khykesko@uci.edu (K.K.); hanau@uci.edu (H.U.); michaela@hs.uci.edu (M.A.)

**Keywords:** islet amyloid polypeptide, amyloid, beta cell, diabetes, islet cell transplant, endoplasmic reticulum stress, unfolded protein response, thioredoxin-interacting protein, 4-phenylbutyrate, tauroursodeoxycholic acid, epigallocatechin gallate

## Abstract

The cytotoxic effect of islet amyloid polypeptide (IAPP) misfolding and aggregation has a well-recognized role in the pathogenesis of type 2 diabetes mellitus, mediated by failure of the beta cell’s protein quality control system to rescue the cell from overwhelming proteotoxic stress induced by IAPP aggregates, ultimately leading to apoptosis. A small but growing body of research also links IAPP-mediated proteotoxic stress to the pathogenesis of type 1 diabetes and to the functional decline of transplanted islets. Among the most promising therapeutic approaches under investigation are small organic molecules that may act as direct chemical chaperones to prevent IAPP aggregation, promote the activity of endogenous chaperones, or alter gene networks of the unfolded protein response (UPR) to promote pro-survival rather than pro-apoptotic pathways in response to IAPP-mediated proteotoxic stress. Compounds warranting special attention include 4-phenylbutyrate (PBA), tauroursodeoxycholic acid (TUDCA), and epigallocatechin gallate (EGCG), as each has a growing body of evidence supporting their ability to ameliorate this process, and given that each of these are already known to have good safety profiles in humans, potentially accelerating the timeline to interventional studies. This review explores the evidence for IAPP-mediated proteotoxicity in multiple forms of diabetes, the mechanisms of cytotoxicity at different levels of the cell’s protein quality control systems, how these small organic compounds may act on these processes including new insights on the role of thioredoxin-interacting protein (TXNIP), and the current evidence supporting each of these compounds in mitigating diabetogenesis.

## 1. Introduction

Diabetes mellitus is an increasing burden to human health worldwide, with over 11% of the global adult population having some form of diabetes mellitus [1] and with this figure rapidly rising. Among children, type 1 diabetes mellitus (T1DM) predominates, but the incidence of both T1DM and T2DM in children is rising [2,3]. New strategies that improve beta cell resilience and survival would be of benefit in most forms of diabetes. Children and adults with newly diagnosed T1DM benefit when they have higher residual beta cell mass, prolonging the “honeymoon” period in which insulin needs are lessened [4]. Increasingly, clinicians can identify children and young adults at risk for development of clinical T1DM via antibody screening, but there are no approved therapies to offer patients with multiple T1DM antibodies who have not yet developed evidence of dysglycemia. For those in the intermediate pre-clinical stage of T1DM, the window of opportunity for the only approved therapy (teplizumab, an anti-CD3 monoclonal antibody) is very narrow, and treatment only delays the onset of clinical T1DM rather than preventing it entirely [5]. In adults with T2DM, a subset will eventually progress to beta cell failure leading to insulin dependence, but this is a prolonged process; in contrast, children with T2DM are much more likely to have rapid onset of beta cell failure leading to insulin dependence [6], and current therapies are not very effective at preventing this [7]. Islet cell transplantation is a therapeutic strategy for individuals with brittle type 1 diabetes who cannot safely be managed on usual insulin regimen due to increased risk of hypoglycemia, but it is limited by high rates of functional islet failure after transplantation [8], as well as by the need for immunosuppression [9]. Currently, there are no non-immunosuppressive pharmacologic therapies to augment beta cell survival after transplant.

Islet amyloid polypeptide (IAPP), also known as amylin, is a peptide hormone co-synthesized and secreted in the insulin granules of the pancreatic beta cell alongside insulin [10], and which has physiologic actions in appetite regulation and mild hypoglycemic effect [11,12]. Separate from this physiologic role, human IAPP and its precursors are prone to misfold and form cytotoxic aggregates, ranging from small oligomers that can disrupt organelle and cellular membranes to large amyloid deposits that may disrupt cell–cell signaling and diffusion [13]. The evidence linking IAPP aggregation to beta cell dysfunction and loss in type 2 diabetes mellitus (T2DM) has been well-established over the last several decades [14,15]. In addition to reviewing the established mechanisms of IAPP-related proteotoxicity in T2DM, we will present the body of evidence that IAPP-related pathology is also involved in the pathogenesis of T1DM and functional islet transplant failure. Given that evidence, we also propose that a unified therapeutic strategy targeting IAPP may be beneficial in prevention or early treatment of multiple forms of diabetes, not only T2DM.

A range of compounds have been tested for their ability to modulate IAPP-mediated proteotoxicity, including tauroursodeoxycholic acid (TUDCA) [16,17,18,19,20], 4-phenlybutyrate (PBA) [21,22,23,24,25,26,27], and epigallocatechin gallate (EGCG) [28,29,30]. While other compounds beyond these three are also under study, we have chosen to highlight the literature on these specific compounds due to each having a combination of in vitro and in vivo evidence for their efficacy, as well as each already having good safety data in humans, as this combination of factors lends an opportunity for accelerated translational study. We will explore the mechanisms by which each of these compounds may exert their effect, including by linking the emerging role of TXNIP elevation in IAPP-mediated proteotoxic stress with the evidence that each of these compounds are able to modulate thioredoxin-interacting protein (TXNIP) expression. We will also explore the limitations of these studies and the models employed, and propose future directions for ongoing study.

## 2. IAPP: Physiologic and Pathologic Mechanisms

### 2.1. Synthesis, Folding, Post-Translational Modification, and Secretion of Human IAPP

Islet amyloid polypeptide (IAPP), also known as amylin, is encoded by the IAPP gene on chromosome 12 and is synthesized as preproIAPP. Insulin is encoded by the insulin gene (*INS*) on chromosome 11p15.5 and is synthesized as preproinsulin. PreproIAPP and preproinsulin are translationally inserted into the endoplasmic reticulum (ER), where each amino acid signal sequence is cleaved from the respective N termini. This process yields proIAPP and proinsulin, which then undergo further modification by the Golgi apparatus and post-Golgi secretory granule to produce functional proteins [31,32].

Post-ER processing of both IAPP and insulin involves enzymatic cleavage by the same complement of enzymes, specifically prohormone convertase 2 (PC2), prohormone convertase 1/3 (PC1/3), and carboxypeptidase E (CPE). IAPP requires one additional enzyme for maturation, not required by insulin, which is peptidyglycine alpha-amidating monooxygenase (PAM). After the action of these enzymes, mature IAPP and insulin are co-stored in insulin secretory granules, where they will be co-secreted through exocytosis upon stimulation by elevated glucose levels [33,34].

Mature monomeric IAPP circulates after secretion and has the physiologic function of lowering appetite and inducing a mild hypoglycemic effect. However, both mature IAPP and its precursors are known to form cytotoxic oligomers as well as larger amyloid aggregates [31]. The smaller cytotoxic oligomers in particular may overwhelm the protein quality control systems of the cell and ultimately lead to apoptosis through mechanisms described in the sections to follow.

### 2.2. Human IAPP, but Not Rodent IAPP, Is Cytotoxic and Amyloidogenic

Accumulation of amyloid deposits composed of IAPP in pancreatic islets is characteristic of type-2 diabetes mellitus (T2DM) [35,36]. siRNA-based silencing of proIAPP mRNA transcripts was shown to enhance the survival and function of cultured human islets, in association with decreased amyloid area, increased insulin content, and improved glucose-stimulated insulin secretion [37], demonstrating IAPP aggregation to be a cause, and not merely a byproduct, of beta cell injury.

Rat IAPP (rIAPP) and mouse IAPP (mIAPP) are non-amyloidogenic due to an absence of histidine and presence of proline in what is the human amyloidogenic region, spanning residues 20–29 of human IAPP (hIAPP), although other regions of the peptide may also play a role in determining relative amyloidogenicity [38]. In general, rodents and other animals with non-amyloidogenic IAPP do not spontaneously develop a T2DM phenotype [39]. In contrast, transgenic mice over-expressing hIAPP spontaneously develop diabetes mellitus in association with islet amyloid deposits [40].

IAPP aggregation results in the cytotoxicity and disruption of multiple elements of the cell’s protein quality control system, each of which, when sufficiently overwhelmed, may trigger apoptosis. While there are many different pathways that lead to apoptosis, they ultimately converge on the effector caspases 3, 6, and 7, which are the proteins that directly dismantle various cellular proteins, leading to chromatin condensation, nuclear fragmentation and finally beta cell death [41,42].

### 2.3. Membrane Disruption

Amyloid formation goes through distinct steps, starting with monomers of IAPP or its precursors. This initial step is known as the lag phase, which leads into the formation of oligomeric intermediates, segueing into the growth phase. Oligomers form protofibrils that assemble into fibrils that will reach the saturation phase where no more growth is observed. Smaller oligomeric assemblies are thought to be more cytotoxic than mature amyloid deposits [40], and this is likely mediated by disruption of cellular and organelle membranes by oligomeric IAPP species [43].

It has been demonstrated in vitro that exogenous IAPP can trigger the production of proinflammatory cytokines, such as interleukin-1 beta (IL-1β), which can sensitize the cell to Fas-mediated apoptosis by upregulating the Fas receptor, promoting extrinsic apoptosis [32]. In vivo, though, intracellular pathways leading to intrinsic apoptosis are thought to be more significant. In beta cells from hIAPP transgenic mice and from humans with T2DM, IAPP oligomers were localized to areas of organelle membrane disruption: at distended ER and Golgi membranes, within remnants of disrupted insulin secretory vesicles, and penetrating into mitochondria with damaged inner cristae. Mitochondria that were close to disrupted secretory vesicles were much more likely to be affected. These findings support membrane disruption, and particularly mitochondrial membrane disruption, as a mechanism of IAPP-induced cytotoxicity [14].

### 2.4. Endoplasmic Reticulum Stress and the Unfolded Protein Response

The endoplasmic reticulum (ER) chaperone system is responsible for assisting proteins in correctly folding into their mature structures, preventing them from misfolding or aggregating. If this system becomes stressed by the accumulation of unfolded or misfolded proteins, as may occur with IAPP aggregation, the unfolded protein response (UPR) is triggered [44]. Studies have demonstrated increased ER stress markers in response to increased hIAPP levels in both humans and rodents [45,46,47,48].

The three main proteins involved in UPR activation are inositol-requiring enzyme-1 alpha (IRE1α), protein kinase RNA-like ER kinase (PERK), and activating transcription factor-6 (ATF6). Activation of the UPR affords the ER more resources to manage the excess of misfolded proteins via upregulation of endogenous chaperones, ER-associated degradation (ERAD) proteins (which traffic misfolded proteins to the ubiquitin–proteasome system), and other proteins involved in execution of the UPR, as well as by downregulating synthesis of other proteins to reduce overall protein synthesis demands. However, if UPR signaling becomes prolonged then this indicates a failure of the cell to successfully overcome ER stress, and the UPR system transitions to pro-apoptotic signaling [44].

IRE1α, PERK, and ATF6 are ER membrane resident proteins and are normally maintained in an inactivated state via binding of their ER luminal surface by binding immunoglobulin protein (BiP), also known as 78 kDa glucose-regulated protein (GRP78). BiP itself is an endogenous chaperone that can bind unfolded proteins. Under conditions of ER stress, unfolded proteins compete with IRE1α, PERK, and ATF6 for BiP binding sites, and cause BiP to dissociate from these UPR proteins. The resulting effects on IRE1α and PERK are similar, as each dimerize/oligomerize in the absence of BiP, resulting in their activation via trans-autophosphorylation. In contrast, ATF6 undergoes a more complex activation process. BiP dissociation exposes ATF6’s disulfide bonds, allowing them to become reduced. This triggers translocation of ATF6 to the Golgi, where it undergoes further processing, cleaving its intra-cytosolic (N-terminal) domain and freeing it from the membrane as a soluble ATF6(N) monomer. ATF6(N) then travels to the nucleus to act as a nuclear transcription factor [49].

IRE1α is an RNAse that can degrade ER-localized mRNA transcripts, reducing translational burden, but also activates several pathways of interest via selective splicing of certain mRNA transcripts. IRE1α promotes a signal cascade that results in activation of the c-Jun amino terminal kinase (JNK) pathway. JNK regulates expression of several transcription factors involved in apoptotic signaling. IRE1α also acts in synchrony with ATF6(N) to activate *XBP1*. ATF6(N) promotes transcription of *XBP1*, and IRE1α is required to splice the *XBP1* mRNA before it can undergo translation into the protein product XBP1s [44]. The effects of XBP1s are described further below, when discussing ATF6(N).

PERK phosphorylates its substrate, the alpha subunit of eukaryotic translation initiation factor-2 (eIF2α). In its unphosphorylated form, eIF2 is part of a protein-mRNa complex that is required for cap-dependent translation, the process by which most proteins are translated (so-called cap-dependent due to this complex recognizing the 5′ mRNA cap to initiate translation). Phosphorylation of eIF2α by PERK inhibits the formation of this complex, thereby slowing the build-up of unfolded proteins in the ER. This mechanism is beneficial for short-term relief of ER stress. This translational blockade permits preferential translation of certain mRNA products that can undergo cap-independent translation, including activating transcription factor-4 (ATF4), although eIF2α enhances ATF4 translation via direct mechanisms as well. ATF4 has some pro-survival functions, such as upregulating antioxidative pathways. However, prolonged ATF4 signaling leads to upregulation of pro-apoptotic C/EBP homologous protein (CHOP). CHOP is a major driver of apoptosis by inhibiting the anti-apoptotic protein Bcl-2 and upregulating the pro-apoptotic protein Bim [44].

ATF6(N), along with XBP1s as described above, are nuclear transcription factors that increase transcription of ER chaperones, protein-modification enzymes, and ERAD components to promote trafficking of misfolded proteins to the ubiquitin–proteasome system. Lipid biosynthetic enzymes are also upregulated, to allow for an increase in the size and capacity of the ER. Under controlled regulation, these factors contribute to the pro-survival aspects of the UPR [44]. However, XBP1s can upregulate expression of CHOP [50], and thus can contribute to pro-apoptotic signaling as well. Additionally, XBP1s binds the promoter region of the *IAPP* gene to increase *IAPP* transcription and thus could worsen IAPP-mediated proteotoxic stress under conditions of ER stress [51].

Notably, many of the genetic syndromes that result in early-onset and even neonatal diabetes mellitus are caused by mutations in genes involved in the ER stress/UPR pathways. These include Wolcott-Rallison syndrome (caused by homozygous mutations in *PERK*), Wolfram syndrome and related syndromes (caused by mutations in *WFS1* or *CISD2*, which are involved in maintaining ER calcium homeostasis and down-regulation of ATF6), the syndrome of ataxia, combined cerebellar and peripheral, with hearing loss, and diabetes mellitus (ACPHD) (caused by mutations in *DNAJC3*, which encodes a co-chaperone for BiP), and the syndrome of microcephaly, short stature, and impaired glucose metabolism-2 (MSSGM2) (caused by mutations in *PPP1R15B*, which is a constitutive repressor of eIF2α phosphorylation) [52]. These conditions could be seen as accelerated models of beta cell loss and diabetogenesis due to unrecoverable ER stress.

### 2.5. The Ubiquitin–Proteasome System

The ubiquitin–proteasome system removes misfolded or aggregated proteins through proteolysis. If this system becomes overwhelmed, the cell may be unable to recover from proteotoxic stress such as that induced by IAPP aggregates [33].

Costes et al. [46] found that beta cells from multiple models expressing human IAPP (human beta cells from individuals with type 2 diabetes, beta cells from hIAPP transgenic rats, and INS 832/13 cells transduced with adenoviruses to express hIAPP) had accumulations of undegraded polyubiquitinated proteins, as well as reduced expression of ubiquitin carboxyl-terminal hydrolase L1 (UCH-L1), a de-ubiquitination enzyme that helps to maintain the total pool of ubiquitin available for protein tagging and degradation. This suggests that IAPP aggregates induce ubiquitin–proteasome dysfunction, preventing this system from relieving the proteotoxic stress induced by the IAPP aggregates, contributing to a cycle of dysfunction. Proposed mechanisms for the downregulation of UCH-L1 by IAPP include decreased expression of the *UCHL1* gene via promoter hypermethylation secondary to ER stress, or post-transcriptional/translational modifications induced by oxidative damage, which could be secondary to IAPP-induced mitochondrial membrane disruption leading to leakage of reactive oxygen species [46].

The changes Costes et al. [46] found were associated with increased phosphorylated eIF2α, as an ER stress marker, as well as pro-apoptotic markers including CHOP and cleaved caspase-3. Overexpression of rat IAPP did not lead to the same downstream effects, pointing to a specific role of hIAPP in causing this sequence of events, and thus presumably of IAPP aggregation, as this is the primary distinguishing factor of human vs. rodent IAPP. *UCHL1*-silenced cells proceeded to cell death at a greater frequency when hIAPP was also expressed, thus the effect was not caused by downregulation of UCH-L1 alone. Of interest is the fact that low levels of UCH-L1 expression were found in islets of pancreatic donors with T2DM compared to nondiabetic donors [46].

Taken together, these findings suggest the ubiquitin–proteasome system can become overwhelmed by hIAPP aggregates, compromising an effective ubiquitin–proteasome response to ER stress and leading to redirection toward pro-apoptotic pathways.

### 2.6. The Autophagolysosome System

Autophagy involves the degradation and recycling of damaged cellular components and is required to maintain normal cellular homeostasis of secretory cells, including beta cells. In cells with hindered autophagolysosomal systems, there is an increased potential for the formation of protein aggregates. At the same time, IAPP aggregates can overwhelm even a normally functioning autophagolysosome system, leading to the failure of rescue from proteotoxic stress and thus leading to apoptosis [53].

Kim et al. (2014) [53] found that hIAPP oligomers accumulate in autophagy-deficient cells, and that, in mice, homozygosity but not heterozygosity for hIAPP was sufficient to induce autophagosomal blockade. Heterozygous hIAPP/mIAPP mice with induced autophagy-related gene 7 (*Atg7*) deficiency developed overt diabetes, in association with increased intracellular hIAPP oligomers, reduced beta cell mass, and positive TUNEL staining indicating apoptosis. When no genetic autophagosomal defect was present, heterozygous hIAPP mice did not develop the same findings. However, homozygous hIAPP^+/+^ mice, with no induced genetic autophagosomal defects, had significantly increased oligomer accumulation. This corresponded to an increase in autophagosome numbers in islet cells but not an increase in autophagic activity. Western blot revealed accumulated p62 protein levels [53]. p62 (also known as SQSTM1 or sequestome 1) is a tagging protein that promotes autophagy of polyubiquinated proteins, and is degraded along with its tagged protein when autophagy is successfully completed, a process which requires the autophagosome to merge with the lysosome [54]. An accumulation of p62 therefore indicates failure to degrade the tagged protein, suggesting the blockade of normal autophagolysosomal function by hIAPP aggregates.

Rivera et al. [55] similarly reported that in hIAPP^+/+^ mice, p62 protein levels were increased vs. wild-type mice. They found, however, that p62 mRNA levels were similar between the two groups. Thus, the different protein levels cannot be due to upregulation of p62 synthesis in hIAPP^+/+^ mice, but instead must be from a decrease in p62 degradation, further supporting the hypothesis that hIAPP aggregates lead to autophagy blockade [55].

Kim et al. (2021) [56] demonstrated that an autophagy-enhancing compound reduced IAPP oligomer accumulation and reduced IAPP-induced apoptosis in multiple in vitro models of human beta-like cells, and partially mitigated impaired glycemic profile in hIAPP transgenic mice [56].

These findings indicate that IAPP aggregation induces blockade of the autophagolysosome system, preventing rescue of the cell from IAPP-related proteotoxic stress via autophagy.

### 2.7. Inflammasome Activation

The effect of IL-1β on beta cell function and apoptosis in T1DM has been extensively studied but is less understood in the context of other forms of diabetes. IL-1β is a proinflammatory cytokine that inhibits β-cell function and induces Fas-triggered apoptosis by activation of nuclear factor kappa B (Nf-κB). NF-κB is involved in the regulation of β-cell function but its overproduction inhibits insulin secretion. This nuclear factor can be activated by tumor necrosis factor alpha (TNF-α) or IL-1β. Hyperglycemia increases IL-1β production in β-cells, and IL-1β-producing beta cells were present in pancreatic sections from donors with type 2 diabetes but not from nondiabetic controls, suggesting that IL-1β may play a role in the pathogenesis of T2DM [57,58].

IL-1β is activated via the NLRP3 inflammasome. This inflammasome cascade ultimately activates caspase-1 to cleave pro-IL-1β into its secreted form. Masters et al. employed bone marrow-derived dendritic cells (BMDC) and macrophages (BMDM) as primary IL-1β releasing cells to investigate inflammasome activation. These professional phagocytic cells produce substantial amounts of IL-1β upon uptake of IAPP oligomers. Islets from transgenic hIAPP mice on a high fat diet were stained with thioflavin S and showed co-localization of IL-1β with amyloid deposits. Further staining analysis suggested tissue macrophages co-localized with some but not all of the IL-1β that was present, suggesting that some of the IL-1β was instead released by islet cells. Similar levels of IL-1β were produced by IAPP stimulation compared to those from particulate adjuvants that were previously known to activate the inflammasome. Unaltered TNF-α and IL-6 levels supported the specific effect of IAPP on IL-1β production rather than an increase in general cytokine production. Additionally, inhibition of caspase-1 in BMDCs led to a reduction in IL-1β production, indicating that its release is dependent on phagocytosis [59].

Activation of the UPR can induce inflammasome activation via an IRE1α-mediated pathway. *MicroRNA-17* (*miR-17*) inhibits translation of the thioredoxin-interacting protein (*TXNIP*) mRNA transcript by binding in the 3′-untranslated region. IRE1α cleaves *miR-17*, releasing this inhibition and allowing TXNIP translation to proceed. One of the downstream effects of TXNIP is activation of the NLRP3 inflammasome and thus IL-1β secretion [60].

Conversely, inflammation can activate the UPR. Human beta cells were exposed to several ER stress-inducing agents, and treatment with the cytokines interleukin-1β, tumor necrosis factor-α, and interferon-γ-induced IRE1α/JNK activation, resulting in elevated levels of heat-shock protein 90 (hsp90). Notably, glucotoxic stress and chemically induced ER stress by thapsigargin did not activate hsp90α [17]. In various cell types, different inflammatory cytokines have been shown to upregulate ER stress and the UPR [61]. Thus, inflammation may contribute to overwhelming ER stress that ultimately leads to apoptosis.

### 2.8. Increased IAPP Transcription in Settings of Hyperglycemia and ER Stress, and the Role of TXNIP

Given that IAPP can induce ER stress, it is notable that ER stress can further enhance expression of IAPP [19,20,21,22], as this implies the potential for a feed-forward process in which IAPP expression leads to further ER stress, leading to further IAPP expression, and so on. Both hyperglycemia and ER stress lead to increased levels of TXNIP. TXNIP increases Forkhead box protein A2 (FoxA2) production, which then attaches to the FoxA2 binding site in the promoter region of the *IAPP* gene, thus increasing *IAPP* transcription [31]. Unlike *IAPP*, *INS* is not directly promoted by FoxA2, and TXNIP has been shown to inhibit insulin production by downregulating the insulin transcription factor MAFA [62]. Reciprocally, insulin can repress *TXNIP* [63].

ER stress has been found to increase TXNIP levels, mediated by IRE1α cleaving the inhibitory miR-17 and thus allowing *TXNIP* translation to proceed [60]. Hyperglycemic stimulation greatly enhances transcription of *TXNIP*. Glucose can be directly sensed by the promoter region of *TXNIP* due to a carbohydrate response element (ChoRE). This region is flanked by two nonpalindromic E-boxes that serve as the recognition site for the transcription factor carbohydrate response element binding protein (ChREBP). Without these motifs, glucose-induced transcription of *TXNIP* is not achieved [64,65].

Thus, both hyperglycemia and ER stress initiate the TXNIP/FoxA2-signaling cascade that leads to increased *IAPP* transcription.

In addition to increasing expression of IAPP, elevated TXNIP is associated with several other downstream effects. As previously discussed, this includes activation of the NLRP3 inflammasome and thus IL-1β secretion [60]. TXNIP inhibits thioredoxin, impairing the cell’s ability to respond to an increase in reactive oxygen species [63], such as might occur with IAPP-induced mitochondrial membrane disruption. TXNIP also increases apoptotic markers such as Bax, Bcl2, caspase-3, and cleaved caspase-9 [65]. Therefore, TXNIP appears to be an important mediator of beta cell pathology and apoptosis.

**Figure 1:**
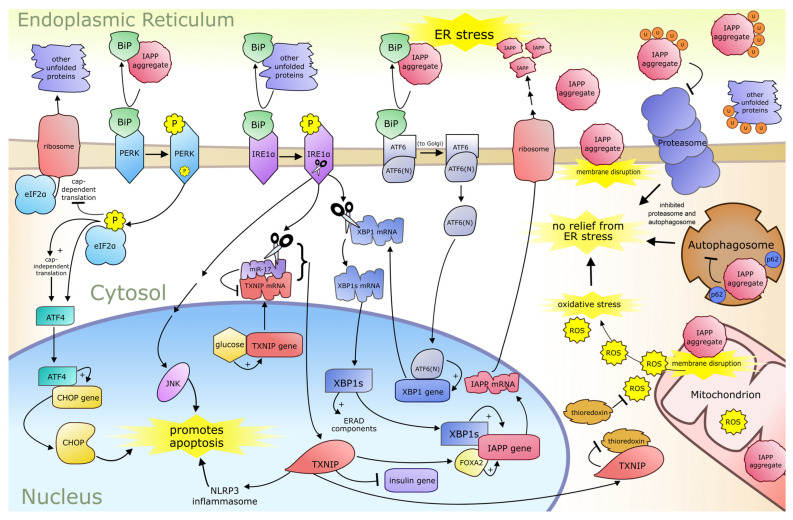
Pathways involved in the self-sustaining cycle of IAPP (insulin amyloid polypeptide)-induced proteotoxic stress. Legend: BiP (binding immunoglobulin protein), PERK (protein kinase RNA-like ER kinase), IRE1α (inositol-requiring enzyme-1 alpha), ATF6 (activating transcription factor-6), ATF6(N) (N-terminal domain of activating transcription factor-6), eIF2α (alpha subunit of eukaryotic translation initiation factor-2), ATF4 (activating transcription factor-4), CHOP (C/EBP homologous protein), JNK (C-jun amino terminal kinase), NLRP3 (NLR family pyrin domain containing 3), *miR-17* (*microRNA-17*), TXNIP (thioredoxin interacting protein), XBP1 (X-box binding protein 1), XBP1s (spliced X-box binding protein 1), ERAD (ER-associated degradation), FoxA2 (Forkhead box protein A2), and p62 (sequestome 1).

Additionally, there is an XBP1s binding site in the *IAPP* promoter region, and thus this is another means by which ER stress upregulates IAPP expression [51]. The combination of glucotoxicity and ER stress can lead to increased *IAPP* transcription and aggregation via increased TXNIP and XBP1s, leading to yet further ER stress, promoting a vicious cycle that if uninterrupted may lead to apoptosis. A summary of IAPP-related pathogenic processes is provided below (Figure 1).

## 3. Role of IAPP in Type 1 Diabetes Mellitus and in Islet Cell Transplantation

### 3.1. In T1DM

T1DM is characterized by autoimmune-mediated beta cell destruction resulting in near-absolute insulin deficiency. As T1DM progresses through several distinct stages in its pathogenesis, when determining if IAPP-mediated processes are involved, we should be mindful that any involvement could be limited to certain phases of disease. If, for example, IAPP cytotoxicity occurs early in T1DM and contributes to the activation of the immune system against pancreatic beta cell antigens, by the time clinical diabetes is apparent, the IAPP-mediated portion of this process may already have largely concluded. Alternatively, IAPP may become involved only once immune-mediated beta cell destruction has already occurred and placed increased synthetic demand on the residual population of beta cells, creating an imbalance of supply and demand that increases ER stress.

While islet amyloid deposits are well-known to occur in T2DM, studies have been somewhat mixed regarding their presence in T1DM. Once beta cells have been lost, further IAPP production from those cells is, of course, halted, and so it is reasonable to expect a limit to the extent of possible amyloid deposition. However, theoretically during the development of type 1 diabetes, and for the variable duration that a residual beta cell population persists, amyloid could be deposited. Westermark et al. [66] examined pancreatic sections from six individuals obtained in the weeks following new diagnosis of type 1 diabetes and found IAPP amyloid deposits in samples from 2/6 patients, compared to 0/5 non-diabetic age-matched control samples. In positive sections, the amyloid deposits clustered both intra- and extracellularly and were associated with dying cells with pyknotic nuclei. Subsequently, Beery et al. [67] demonstrated IAPP amyloid deposits in 3/3 pancreatic samples from deceased donors with type 1 diabetes, compared to 0/3 for matched non-diabetic controls [67]. This evidence suggests that IAPP-related pathology is associated with at least some patients with type 1 diabetes, but this conclusion is limited by small sample size.

Paulsson et al. [34] examined whether levels of plasma IAPP were increased in pediatric patients with newly diagnosed type 1 diabetes. Increased levels were found in a fraction of patients studied (11%), some markedly so. Higher levels of IAPP did not correlate with levels of C-peptide or proinsulin [34], which could be due to differential transcriptional regulation of IAPP vs. insulin. Courtade et al. [68] found that the ratio of proIAPP to mature IAPP was increased in children with longstanding type 1 diabetes (as well as in islet transplant recipients). The absolute level of both proIAPP and mature IAPP was reduced relative to the general population, as expected given the loss of beta cells during disease progression [68]. The altered ratio of proIAPP to IAPP thus seems to represent dysfunctional post-translational processing in the residual beta cell population. This has been well-characterized for insulin and its processing intermediates, as well [69]: Sims et al. (2016) [70] found an elevated ratio of proinsulin to C-peptide in pediatric patients with new-onset type 1 diabetes, and that an elevated ratio increased the risk that autoantibody-positive patients would progress to clinical type 1 diabetes [70]. This suggests a role of altered prohormone convertase processing in the pathogenesis of type 1 diabetes. IAPP-induced stresses on the protein processing machinery could be an explanatory mechanism for this finding. It is not known if IAPP overexpression or aggregation directly alters prohormone convertase levels, nor whether accumulation of IAPP precursors can competitively limit the availability of prohormone convertases for processing of insulin precursors. ER stress itself, though, has been shown to reduce the expression of prohormone convertases [71,72].

Additionally, Sims et al. (2018) [73] found that 90% of individuals with longstanding type 1 diabetes continue to have detectable proinsulin levels even when C-peptide is undetectable, suggesting that part of the decline of C-peptide over time is due to dysfunctional prohormone convertase activity in residual beta cells rather than being entirely from beta cell death [73]. If patients with longstanding type 1 diabetes frequently have residual but dysfunctional beta cell populations, and if at least some of this dysfunction is caused by IAPP aggregation and ER stress, then targeting these mechanisms could salvage some residual function. This could mitigate the severity of clinical type 1 diabetes, reduce the amount of exogenous insulin needed to maintain good glycemic control, and reduce the risk of dysglycemic excursions.

It remains unclear whether there is an increased prevalence of anti-IAPP antibodies in type 1 diabetes. Studies using different assays to measure anti-IAPP antibodies have reached differing conclusions. Using an ELISA-based assay, Clark et al. [74] found elevated anti-IAPP antibody levels in 18% of samples from patients with type 1 diabetes, relative to a standardized non-diabetic control serum [74]. In contrast, Gorus et al. [75] developed a radiobinding assay to detect anti-IAPP antibodies and found no increased prevalence of such antibodies in patients with type 1 diabetes when compared to the general population. The authors discussed two notable limitations of this finding: first, that their assay might not detect antibodies directed against the C-terminal section of IAPP, and second, that their assay would only be expected to detect monomeric IAPP [75]. Given that it is the oligomeric species that are cytotoxic, it remains unknown if there are antibodies against IAPP oligomers involved in the pathogenesis of type 1 diabetes.

In contrast to these unclear findings regarding IAPP antibodies, multiple studies have found that IAPP or its processing intermediates can act as T-cell epitopes in both humans with T1DM and in mouse models of T1DM. In humans this has been demonstrated for the common HLA class I allele, HLA-A*0201 (which confers some additional risk of type 1 diabetes in individuals with certain high-risk HLA class II alleles), but has not yet been studied in patients with type 1 diabetes with other haplotypes [76,77,78].

Proteotoxic stress from IAPP aggregation may increase the amount of immature insulin processing intermediates present, with proinsulin and preproinsulin better-known to be immunogenic antigens and epitopes in type 1 diabetes [79,80]. Additionally, hybrid insulin peptides (HIPs) are fusion peptides combining fragments of processing intermediates of insulin with fragments of other beta cell proteins, including IAPP and its precursors, and these have been discovered as T-cell antigens in diabetic mouse models [81], in cadaveric islets from patients with T1DM [82], and in patients with newly diagnosed T1DM [83]. HIPs derived from C-peptide and IAPP have also been found as T-cell epitopes in individuals at high genetic risk for developing T1DM, particularly among those who eventually went on to develop clinical T1DM [84,85]. Thus, impairments in IAPP and insulin processing (which could be induced by IAPP aggregation and resulting ER stress) could be a risk factor for the development of T1DM.

### 3.2. In Islet Cell Transplantation

Islet transplantation via infusion of allogenic donor islets into the portal vein is an FDA-approved treatment for adults with fragile type 1 diabetes who experience severe recurrent hypoglycemia with usual treatment with exogenous insulin. However, in addition to requiring lifelong immunosuppression to prevent allogenic graft rejection, this procedure is limited by the small numbers of viable islets that survive the retrieval and transplantation process. The small number of transplanted beta cells must then do the protein synthetic work that would normally be accomplished by a much larger population of beta cells in a non-diabetic individual, subjecting each cell to greater synthetic stress. Oxygen supply to transplanted islets is limited until revascularization occurs, further increasing the stress on these cells. Even when immunologic rejection does not occur, islet transplants are prone to functional failure over time, where the transplanted beta cells become unable to meet the patient’s insulin needs and the patient once more becomes dependent on exogenous insulin administration, or requires repeat transplantation [86,87]. It is possible that IAPP-induced cytotoxicity mediates this process of transplanted-beta-cell decline.

Human donor islets transplanted into nude mice rapidly developed both intracellular and extracellular amyloid deposits within 2 weeks of transplant [88]. When the donor islets were human in origin, intracellular deposits predominated, whereas if the donor islets were from transgenic hIAPP-expressing mice, extracellular deposits predominated. The reason for this difference is unclear, but points to the need for studies in human cells/tissues and not only in hIAPP transgenic rodents [89]. In non-transgenic streptozocin-induced diabetic mice receiving donor islets from hIAPP transgenic mice, amyloid developed in the vast majority of grafts by 6 weeks post-transplant, was associated with beta cell apoptosis, and preceded the recurrence of hyperglycemia [90].

Like humans, non-human primates have amyloidogenic IAPP. Islets from normoglycemic donor macaques were transplanted into recipient macaques with streptozocin-induced diabetes. Animals with early immune-mediated graft rejection had not accumulated islet amyloid at the time of the graft failure. In contrast, animals with successful transplants without immune-mediated rejection gradually accumulated increasing islet amyloid over time, associated with decreasing beta cell area, followed by recurrence of hyperglycemia [91].

In humans, in several post-mortem studies of islet transplant recipients who died of other causes, biopsies detected frequent IAPP-positive amyloid deposits in the transplanted islets of four out of five of these patients. Certainly, this finding is limited by the small sample size and lack of controls. It is notable, though, that the only patient lacking these deposits had the lowest hemoglobin A1c of the group and had remained insulin-independent by the time of their death [92,93].

In combination, these studies suggest that IAPP-mediated cytotoxicity likely contributes to the functional decline of transplanted beta cells.

## 4. Small Organic Molecules as Chaperones and Modulators of ER Stress

The ER possesses a wide variety of endogenous chaperone proteins that sustain proteostasis by decreasing the accumulation of misfolded and aggregated proteins through effects on protein synthesis, folding, and degradation. These include various heat shock proteins, protein disulfide isomerase (PDI), BiP, and more [94]. Some assist only a limited species of proteins in achieving proper folding, while others are more nonspecific. Chemical chaperones are compounds that help achieve this same task and may be of particular use in scenarios where the endogenous chaperone system would otherwise become overwhelmed [95]. By improving the ER’s capacity for protein folding and influencing signaling through the unfolded protein response (UPR), chemical chaperones contribute to reducing misfolded protein burden and alleviating ER stress [96]. Phenolic compounds such as 4-phenylbutyrate (PBA) and epigallocatechin gallate (EGCG) appear to stabilize misfolded proteins nonspecifically via hydrophobic interactions [23,30]. Some compounds, such as tauroursodeoxycholic acid (TUDCA), have been proposed as chemical chaperones due to their ability to relieve ER stress. However, evidence for TUDCA’s direct interactions with target proteins, such as misfolded IAPP, is less robust and these are now thought to act primarily through indirect modulation of signaling pathways, theoretically allowing the endogenous chaperone system to work more effectively [97]. Once the UPR is activated, both direct chemical chaperones and chemical modulators of endogenous chaperones can help to limit the accumulation of misfolded proteins, supporting the maintenance of the adaptive, pro-survival phase of the UPR and reducing the likelihood of transition to a persistent, pro-apoptotic response [98].

### 4.1. TUDCA

TUDCA is the taurine-conjugated derivative of ursodeoxycholic acid (UDCA), a compound that is FDA-approved for the treatment of primary biliary cholangitis and other cholestatic liver disorders. It is produced at low levels endogenously. Although TUDCA shares UDCA’s favorable safety profile, its mechanism of action in improving cell survival has not been fully defined and is likely multimodal. TUDCA has been proposed as a chemical chaperone, but evidence for this is limited. In a cell-free assay, it was shown to reduce aggregation of denatured bovine serum albumin (BSA), thus there is the potential for direct chaperone activity [99]. Unlike PBA and EGCG, though, TUDCA has not been tested for its ability to disaggregate IAPP, specifically, in cell-free assay. Instead, most research into TUDCA’s ability to mediate ER stress has explored its indirect effects via altered signaling pathways. As an example, in a hepatocyte model, TUDCA has been reported to promote survival through decreased activation of the PERK–eIF2α–ATF4–CHOP arm of the UPR [97]. In addition to indirectly modulating the UPR system, there is evidence supporting other mechanisms of action that could contribute to a globally improved glycemic profile.

TUDCA and other bile salts are known to be transported intracellularly via several classes of transporters. Some of these are restricted to hepatocytes, but some are also expressed by beta cells, including certain organic anion-transporting polypeptides (OATPs) such as OATP1B3 [100,101]. It is not well understood whether TUDCA’s benefits to pancreatic beta cells arise from intracellular vs. extracellular effects, or both. In a study of porcine embryos exposed to glucotoxic stress, extracellular TUDCA reduced ER stress and improved survival, whereas knockdown of the bile acid receptor Takeda G-protein-coupled receptor 5 (TGR5) abolished these protective effects [102]. TGR5 is a G-protein-coupled receptor with tissue-dependent effects, with taurine conjugation enhancing the affinity of bile acid ligands for the receptor. It is controversial whether TGR5 internalizes after activation or remains on the cell membrane, as different groups have reported conflicting findings [103,104]. Downstream signaling of TGR5 is tissue-dependent, and in some cell types has been shown to modulate ER stress pathways. Additionally, stimulation of TGR5 receptors on enteroendocrine cells causes release of GLP-1, thus has the potential to improve systemic glycemic functioning separate from direct effects on beta cells [105]. In beta cells, TGR5 has been shown to potentiate insulin release by stimulating a rise in intracellular calcium [106,107]. Another bile acid receptor, the farnesoid X receptor (FXR), is also activated by TUDCA and stimulates beta cells to increase synthesis of insulin. FXR activation induces Akt phosphorylation, which decreases NF-κB, 1L-1β, and caspase-3, thus promoting cell survival [108,109].

Stimulation of FXR has been shown to reduce ER stress markers in mouse hepatocytes, in addition to reducing activation of TXNIP and the NLRP3 inflammasome [110]. FXR was found to inhibit ChREBP, preventing its interaction with the ChoRE in the promoter region of *TXNIP* [111]. It is possible this could account for the finding in multiple studies that TUDCA reduces expression of TXNIP [112,113,114], including in beta cells [75].

FXR activation increased expression of PC 1/3 in mouse ileal cells, but it is unknown if it has this effect in beta cells [115]. If so, this could contribute to TUDCA’s ability to mitigate beta cell ER stress by increasing the pool of processing enzymes that contribute to formation of both mature IAPP and mature insulin.

Additional actions of TUDCA have been linked to the regulation of inflammatory pathways. By reducing NF-κB activation and the downstream release of pro-inflammatory cytokines such as TNF-α, IL-1β, and IL-6, TUDCA dampens inflammatory signaling that otherwise could exacerbate ER stress [116]. Chronic inflammation is known to promote oxidative stress and disrupt calcium homeostasis, both of which contribute to protein misfolding and sustained UPR activation. By attenuating this inflammatory feedback loop, TUDCA supports the adaptive phase of the UPR, increasing protein-folding capacity and promoting cell survival [117,118].

Bronczek et al. [16] utilized a streptozotocin (STZ)-induced diabetes mouse model and Engin et al. [19] utilized two autoimmune diabetic mouse models to investigate the effect of TUDCA on diabetogenesis in these models. STZ chemically induces beta cell death (variably via necrosis or apoptosis depending on dosage and timing of STZ administration) leading to insulin-deficient diabetes, without involvement of the adaptive immune system. Autoimmune mouse models (the NOD mouse model being the most common) more closely reflect the underlying pathophysiology of human type 1 diabetes via self-T-cell-mediated apoptotic death of beta cells. Both groups found significant improvements in diabetes development or progression when given TUDCA [16,19], indicating that TUDCA’s benefits do not exclusively arise from interactions with IAPP folding dynamics, as none of the models were transgenic for hIAPP and thus expressed non-amyloidogenic wild-type mouse IAPP. However, studies utilizing models without human IAPP may still be instructive in clarifying TUDCA’s role in mitigating ER stress more generally. Several studies have examined the administration of TUDCA to transgenic mice expressing hIAPP, but in these cases 4-phenybutyrate (PBA) was trialed as well, and so these studies are discussed in a later section.

In Bronczek et al. [16] oral TUDCA was administered only after induction of diabetes and the STZ + TUDCA group demonstrated a 43% reduction in blood glucose compared to the STZ-only group, with increased beta cell mass and numbers. Hepatic insulin-degrading enzyme (IDE) activity was significantly lower in the STZ + TUDCA group, suggesting another possible mechanism for improved glycemic control [16].

In Engin et al. [19] two autoimmune mouse models were used, the NOD mouse model as well as the RIP-LCMV-GP (rat insulin promoter-lymphocytic choriomeningitis virus-glycoprotein) model. A decrease in expression of ATF6 was noted in both models upon emergence of diabetes. TUDCA was administered via intraperitoneal injections in the prediabetic stage and led to marked reduction in diabetes incidence in the treated mice. This benefit was lost in mice with β cell-specific deletions of *ATF6*, indicating that at least part of TUDCA’s benefit requires functional ATF6 activity. TUDCA-treated NOD mice demonstrated less insulitis, despite similar pancreatic and circulating numbers and types of lymphocytes. Additionally, the group demonstrated reduced ATF6 and sXBP1 activity in the beta cells of human pancreatic sections from donors with T1DM when compared to non-diabetic controls [19].

This relationship would seem to stand in contrast to findings by several groups that TUDCA decreases ATF6 expression, along with other markers of UPR stress [119]. This apparent contradiction might be explained by a dose–response relationship of the UPR itself, where mild activation of the UPR promotes chaperone activity and permits rescue from ER stress, while ongoing UPR activation indicates failure of rescue and promotes pro-apoptotic pathways. Thus, TUDCA might upregulate ATF6 under some circumstances and downregulate it in others. In human beta cells, a pro-inflammatory cytokine milieu has been shown to induce UPR signaling and subsequently pro-apoptotic signals, via IRE1-induced activation of JNK and subsequently hsp90α. Brozzi et al. [18] and Ocaña et al. [17] demonstrated that TUDCA could inhibit JNK activation and hsp90α in response to cytokine stress in human beta cells in vitro [17,18]. Although persistently elevated ATF6 can also promote pro-apoptotic pathways downstream, it is the main transcription factor to upregulate chaperone protein synthesis during UPR stress, and Walter et al. [120] demonstrated that loss of ATF6 expression leads to uncontrolled IRE1 signaling which is more strongly associated with apoptosis [120].

TUDCA has been studied in humans with an overall good tolerability profile. Kars et al. [20] administered 4 weeks of oral TUDCA or placebo to a group of obese but nondiabetic participants. Markers of ER stress in muscle and adipose tissue did not change, but pancreatic/beta cell ER stress was not assessed. Hepatic and muscle insulin sensitivity was improved [20].

### 4.2. PBA

PBA is a small aromatic fatty acid that is FDA-approved as an ammonia scavenger for the treatment of urea cycle disorders [121]. Acting as a direct chemical chaperone, PBA stabilizes folding intermediates and reduces insoluble protein aggregation by binding to exposed hydrophobic patches on misfolded proteins [23,95]. In mammalian models, PBA has been shown to improve ER folding capacity and reduce apoptosis in multiple cell types. In human renal proximal tubular cells in vitro, and murine renal tissues in vivo, PBA administration was protective against apoptosis and was associated with lower levels of CHOP and BiP [122]. In cardiac cells exposed to excess palmitic acid, PBA reduced expression of ER stress and apoptotic markers including CHOP, BiP, BAX, and caspase-3 [123]. Multiple studies show that PBA can lower levels of TXNIP expression in various tissues [23,112,124,125].

A secondary mechanism by which PBA can reduce ER stress is by acting as a histone deacetylase (HDAC) inhibitor, although the mechanisms and cell-specificity of this finding are not yet well-understood. HDACs increase histone acetylation, resulting in a more relaxed chromatin structure and enhanced transcription of genes, thus the net effect of an HDAC inhibitor depends on which genes are preferentially expressed and in what context, with the potential for both beneficial and deleterious effects [126]. As an example, in skeletal myocytes PBA enhances expression of GLUT4, the primary transporter that carries glucose into myocytes in an insulin-dependent manner, thus improving peripheral insulin sensitivity. This was found to occur independent of its direct ability to modulate ER stress [127]. Downstream, it is possible this could reduce beta cell ER stress indirectly by reducing hyperglycemic stress.

Montane et al. [23] investigated the interactions of PBA with IAPP across multiple model types. They used computational modeling to show that PBA is predicted to bind to hIAPP at specific binding sites and confirmed this via thioflavin T assay and electron microscopy showing PBA preventing fibril formation in cell-free media, in a dose-dependent manner. This group also administered oral PBA to transgenic mice engineered to overexpress hIAPP and found that giving PBA restored normoglycemia. Specifically, PBA was administered starting once mice were 8 weeks old; at this point mice were demonstrating impaired glucose tolerance. After 12 weeks of treatment with PBA or placebo, placebo-treated mice had worsening dysglycemia, whereas PBA-treated mice had glucose tolerance tests and glucose-stimulated insulin secretion resembling WT controls. Additionally, hIAPP transgenic mice had upregulation of genes related to inflammation, including elevated IL-1β and TXNIP expression, and this gene expression profile was largely normalized by PBA treatment. Mouse hIAPP islets cultured under glucolipotoxic conditions had impairment in insulin secretion and insulin content and had significant elevation in percent of cleaved caspase-3 positive cells. hIAPP islets also rapidly developed amyloid deposition. These changes did not occur in hIAPP islets cultured with PBA. Additionally, pre-existing islet amyloid deposition induced by 7 days of glucolipotoxic conditions could be reversed with 24 h of PBA exposure [23].

de Pablo et al. [22] used agouti viable yellow mice (a strain predisposed to obesity and insulin resistance) crossed with hIAPP-expressing mice and found that they developed overt diabetes along with islet amyloid deposition, and this was greatly ameliorated by administration of oral PBA starting once mice began showing impaired glycemic function at 8 weeks of age, with notable improvements in fasting and postprandial glycemia in the PBA-treated group, along with reduced islet amyloid. PBA treatment did not completely normalize glycemic function, likely owing to inherent severe insulin resistance in the agouti viable yellow phenotype [22].

In humans, Xiao et al. [26] administered oral PBA for two weeks to overweight or obese but nondiabetic participants, and found that it partially alleviated lipid-induced insulin resistance [26]. Vanweert et al. [21] administered oral PBA to patients with T2DM in a randomized, placebo-controlled, double-blind crossover study, with results showing improved peripheral insulin sensitivity versus placebo, along with several other measures of metabolic functioning. Notably, there were no adverse effects observed in either study [21].

Thus, like TUDCA, PBA may have additional systemic effects that could mitigate diabetogenesis, beyond its ability to act on IAPP-mediated processes. Research clarifying the relative role of these different mechanisms is needed.

### 4.3. Both TUDCA and PBA

Several studies have simultaneously studied TUDCA and PBA in the context of diabetes and/or IAPP. Cadavez et al. [24] examined a rat beta cell line engineered to express hIAPP, exposing the cells to either thapsigargin to induce chemical ER stress, or to glucolipotoxic stress. These cells showed increased expression of UPR and apoptotic markers including ATF3, spliced XBP1, phosphorylated eIF2α, CHOP, and caspase-3, versus cells expressing rat IAPP. Treatment with either TUDCA or PBA relieved this increase in UPR and apoptotic markers, and improved glucose-stimulated insulin secretion. Similar benefits were found by overexpressing endogenous chaperones BiP or PDI [24].

Özcan et al. [27] used a leptin-deficient ob/ob mouse model, another model predisposed to obesity and insulin resistance. Untreated, these mice developed marked hyperglycemia and abnormal glucose tolerance, as well as increased liver and adipose PERK, IRE-1α, and JNK activation. When treated with oral PBA, these findings normalized and instead resembled that of the wild-type controls. Beta cell ER stress markers were not examined, nor did they use mice expressing hIAPP, but their findings still demonstrate the ability of PBA and TUDCA to moderate ER stress and diabetogenesis more generally [27].

Shang et al. [25] developed an induced pluripotent stem cell (iPS)-derived human beta cell model, derived from samples from patients with diabetes secondary to Wolfram syndrome, caused by mutations in *WFS1*. A subset of these were transfected to restore wild-type *WFS1* to act as controls, as well as iPS-derived beta cells from control subjects without Wolfram syndrome. At baseline, ER stress markers were increased in Wolfram-derived cells, as expected given that WFS1 is involved in maintenance of the ER calcium gradient. Exposure to chemically induced ER stress amplified this difference versus control cells, whereas treatment with either PBA or TUDCA normalized ER stress markers and improved insulin secretory capacity. Thus, in a human beta cell model with intrinsic increased vulnerability to ER stress, PBA and TUDCA were effective in ameliorating this stress [25]. Kitamura et al. [72] utilized a similar model but gave TUDCA and PBA in combination, and this inhibited apoptosis and restored insulin secretion in vitro.

### 4.4. EGCG

Epigallocatechin gallate (EGCG) is a natural polyphenolic compound found in green tea. Studies have linked it to numerous proposed health benefits, and in beta cells lines in vitro it has been associated with lower CHOP levels and reduced apoptosis [128,129]. Though the relationship between EGCG and TXNIP levels has not examined in beta cells, EGCG was found to reduce TXNIP levels in podocytes in a mouse model of diabetic kidney disease [130]. EGCG has demonstrated a marked ability to not only prevent IAPP aggregation in vitro, but also actively disaggregate pre-formed IAPP aggregates, demonstrated both on thioflavin T (ThT) fluorescence curves and on electron microscopy [29,30]. Thus, EGCG likely acts as a direct chemical chaperone, assisting IAPP in refolding into a soluble form. Studies in vivo are limited, however. Franko et al. [28] investigated treatment of hIAPP transgenic mice with EGCG. Both heterozygous hIAPP/mIAPP as well as homozygous hIAPP mice were compared to homozygous wild-type mIAPP controls. Without EGCG treatment, homozygous hIAPP mice had markedly elevated blood glucose levels and decreased fed insulin levels relative to controls, associated with decreased islet numbers and disrupted islet structures. However, heterozygous hIAPP/mIAPP mice had no significant differences from homozygous mIAPP controls in metabolic measurements, and only mild changes to islet morphology. With 3 weeks of EGCG treatment, the changes seen in homozygous hIAPP mice were not significantly improved. There was some decrease in amyloid fibril intensity in the islets of heterozygous mice [28]. Thus, despite possessing an impressive ability to inhibit and reverse IAPP aggregation in vitro, EGCG’s appeared to underperform in vivo. However, EGCG was administered only after overt diabetes and beta cell loss had already developed, and it is possible it may be more efficacious as a preventive measure earlier in the disease course. This may account for the underperformance of EGCG in vivo, and thus EGCG should remain a candidate compound of interest. As of yet, we are not aware of any studies of hIAPP-expressing models in which EGCG was given preventively. A summary of compounds with efficacy on IAPP and their relevant studies is provided below (Table 1).

### 4.5. Other Small Organic Molecules

A number of other small, naturally occurring compounds have shown promise in inhibiting IAPP aggregation in vitro. These are largely small phenolic compounds and include morin hydrate [131], resveratrol [132], and the extract of Washingtonia filifera seed, which are largely composed of a mixture of different flavanols [133]. However, none of these have been tested for in vivo efficacy against IAPP.

## 5. Conclusions and Future Directions

Islet amyloid polypeptide (IAPP) aggregation can induce significant proteotoxic stress in pancreatic beta cells, which can lead to beta cell apoptosis and ultimately diabetes. This is mediated by: (1) endoplasmic reticulum (ER) stress from accumulation of misfolded IAPP, (2) disruption of organelle membranes including mitochondrial membranes leading to leakage of reactive oxygen species, (3) the ability of IAPP aggregates to blockade the systems cells use to directly reduce proteotoxic stress, namely, the proteasome and the autophagolysosome, and (4) the tendency for the cell’s unfolded protein response (UPR), which is activated by these combined stresses, to further increase expression of IAPP. These factors contribute to a self-sustaining cycle of IAPP-induced proteotoxic stress, and if the cell is unable to overcome this cycle, eventually triggers a shift from pro-adaptogenic to pro-apoptotic pathways of the UPR.

UPR activation leads to increased expression of IAPP via spliced X-box binding protein 1 (XBP1s) and thioredoxin-interacting protein (TXNIP), the latter of which is also activated by hyperglycemia. TXNIP additionally impairs the cell’s response to oxidative stress, activates pro-inflammatory pathways, and stimulates the intrinsic mitochondrial pathway of apoptosis. This makes TXNIP a potentially attractive target for therapeutic modulation.

IAPP-mediated dysfunction is well-characterized in the pathogenesis of type 2 diabetes mellitus (T2DM). An essentially similar pathologic process is expected whenever there is greatly increased protein synthetic demand on individual beta cells, such as in islet transplantation and in the late stages of development of type 1 diabetes mellitus (T1DM), that is, once immune-mediated beta cell losses progress to the point that the remaining beta cell population cannot adequately meet an individual’s insulin needs. In T1DM this suggests that IAPP aggregation might accelerate beta cell losses once autoimmune attack has begun, but it is less clear whether IAPP contributes to developing beta cell-directed autoimmunity in the first place. There is only mixed and low-quality evidence for anti-IAPP antibodies in T1DM. On the other hand, several studies have demonstrated that IAPP or its precursors, as well as proIAPP-containing fusion peptides, act as a T-cell epitope in humans with T1DM (at least in certain haplotypes) and in mouse T1DM models. Dysfunctional beta cells tend to secrete an increased ratio of proinsulin to insulin, and proinsulin is thought to be more immunogenic than mature insulin. Similarly there is an increased ratio of proIAPP to IAPP in T1DM. Insulin and IAPP largely share the same protein processing enzymes to achieve their mature forms, but it is not known whether IAPP overexpression or aggregation might competitively inhibit insulin precursors from being processed by this machinery. ER stress is known to decrease expression of these processing enzymes, however, and so IAPP-induced stresses may increase the proinsulin-to-insulin ratio and thus indirectly could increase beta cell autoimmunity, even if IAPP itself is not a direct antigen or epitope.

As there is evidence for the role of IAPP-induced proteotoxic stress in all major forms of diabetes, therapies that directly target IAPP aggregation or that promote pro-adaptogenic arms of the unfolded protein response (UPR) are worth further investigation as these might improve general beta cell resilience to multiple stressors. By enhancing beta cell resilience and survival, such a therapy could be used to prevent or delay progression during the preclinical stages of T1DM, decrease insulin needs and extend the honeymoon period in clinical T1DM, improve functional islet transplant outcomes, and/or prevent or delay progression to insulin dependence in T2DM. No approved therapy currently addresses these needs.

Currently, compounds showing the most promise in in vitro studies, as well as in in vivo animal studies, are tauroursodeoxycholic acid (TUDCA) and 4-phenylbutyrate (PBA). Epigallocatechin gallate (EGCG) is another compound that is quite promising, with marked ability to disaggregate IAPP in vitro but with ambiguous in vivo results, although this difference may be due to study design in which EGCG was given only after significant beta cell loss had already occurred. Each of these compounds has been shown to reduce levels of TXNIP, although for EGCG this has not been studied in beta cells specifically. These three compounds additionally have the benefit of known good safety profiles in humans, potentially accelerating a timeline to interventional studies.

Mechanistically, PBA and EGCG likely act as direct chemical chaperones to inhibit and even reverse IAPP aggregation. PBA has additional actions as an HDAC inhibitor, leading to changes in gene expression networks. There is evidence TUDCA can act as a direct chemical chaperone for some proteins, though this has not been studied for IAPP specifically. Instead, TUDCA may act via bile acid receptors such as Takeda G-protein-coupled receptor 5 (TGR5) and the farnesoid X receptor (FXR) to exert downstream effects on the UPR to promote survival rather than apoptosis. FXR activation has been shown to induce transcriptional repression of *TXNIP*, and so it is possible that this is the most pertinent mechanism by which TUDCA relieves IAPP-induced beta cell stress.

These compounds also appear to act on non-beta cell targets and improve systemic metabolic function more globally, such as improved peripheral insulin sensitivity, based on studies in other cell types and based on in vivo studies in non-diabetic humans. While this could be a boon to overall efficacy of these compounds when given to patients, it will be important to clarify which effects are related vs. unrelated to interactions with IAPP aggregation. While studies of transgenic rodents expressing hIAPP have yielded important insights, there are other important differences in human vs. rodent beta cells that ultimately limit our ability to draw conclusions. In vitro studies of these compounds in human cell lines have been relatively few and could yield important differences from transgenic rodent studies. Should future studies continue to be promising, then ultimately human clinical trials would be needed to determine whether any of these compounds have a role in the clinical treatment of diabetes.

## Figures and Tables

**Table 1:** Summary of compounds targeting the IAPP pathway and their relevant studies.

Study	Compound(s)	Model	hIAPP	Administration	Outcome(s)
Bronczek et al. [16]	TUDCA	Mouse C57Bl/6 strain with STZ-induced diabetes, in vivo	No	300 mg/kg/day TUDCA	Fasting glycemia reduced by 43% in 2 weeks Improved glucose tolerance Increased plasma insulin levels in fed state Increased β-cell number and mass
Engin et al. [19]	TUDCA	Mouse NOD and RIP-LCMV-GP strains with autoimmune diabetes, in vivo	No	250 mg/kg 2× daily TUDCA	Dose-dependent decrease in incidence of T1D Preserved insulin secretion Improved β-cell morphology Restored ATF6 levels Reduced apoptosis
Chan et al. [114]	TUDCA	Mice exposed to 2 wk angiotensin II infusion, in vivo	No	150 mg/kg/day TUDCA	Improved glucose tolerance In islets isolated from treated animals, normalization of elevated levels of ER stress markers including TXNIP
Ocaña et al. [17]	TUDCA	Human beta cell line (*β*Lox5) with cytokine-induced stress	Yes	0.2 or 1 mM TUDCA for 6 h	Inhibited hsp90*α* release in response to cytokine stress Partially blocked up-regulation of ATF3-inhibited JNK phosphorylation at 30 min and 24 h
Brozzi et al. [18]	TUDCA	Human beta cells with IL-1β, TNF-α, IFN-γ -induced stress	Yes	62 or 125 μmol/L for 2 or 24 h TUDCA	Reduced cytokine-induced apoptosis Lowered CHOP expression Prevented JNK pathway activation
Kars et al. [20]	TUDCA	Obese, non-diabetic human participants	Yes	4 weeks, oral 1750 mg/day TUDCA	Improved hepatic and muscle insulin sensitivity
de Pablo et al. [22]	PBA	hIAPP transgenic mice x obese agouti viable yellow, in vivo	Yes	1 g/kg/day PBA for 12 weeks	Improved fasting and postprandial glycemia Reduced islet amyloid deposits
Montane et al. [23]	PBA	hIAPP transgenic mice, in vivo	Yes	1 g/kg/d PBA dissolved in drinking water for 12 wk, starting at 8 wk of age	Normalized glucose tolerance tests Restored glucose-stimulated insulin secretion Normalization of gene expression profiles, including of IL-1β and TXNIP
		hIAPP transgenic mouse islets in glucolipotoxic culture conditions	Yes	2.5 mM PBA dissolved in PBS	Restored glucose-stimulated insulin secretion Abolished activation of caspase-3 Prevented islet amyloid deposition Reversed pre-existing islet amyloid deposition
		In silico analysis	Yes	Crystalline structure of PBA and hIAPP examined	PBA predicted to bind hIAPP aggregates of various sizes
		Cell-free culture with 500 mM hIAPP	Yes	2.5, 5, and 25 mM PBA	Prevented fibril formation in dose-dependent manner as detected by thioflavin T fluorescence (TfT) and transmission electron microscopy (TEM)
Vanweert et al. [21]	PBA	Human participants with T2DM	Yes	Oral PBA 4.8 g/m^2^/day	Improved peripheral insulin sensitivity Elevated mitochondrial oxidative capacity
Xiao et al. [26]	PBA	Obese, non-diabetic human participants	Yes	Oral PBA 7.5 g/day for 2 weeks	Partially alleviated lipid-induced insulin resistance
Cadavez et al. [24]	TUDCA + PBA	Rat beta cell line (INS1E) expressing hIAPP, exposed to thapsigargin or glucolipotoxic stress	Yes	200 uM TUDCA, 2.5 mM PBA, for 24 h	Reduced CHOP, ATF3, and p-eIF2α levels Improved glucose stimulated insulin secretion
Shang et al. [25]	TUDCA + PBA	iPS-derived beta cells from patients with Wolfram Syndrome	Yes	1 mmol/L PBA or 1 mmol/L TUDCA 1 h prior and during thapsigargin treatment	Reduced CHOP and BiP/GRP78 levels Improved insulin secretion Decreased apoptosis rates
Kitamura et al. [112]	TUDCA + PBA	iPS-derived beta cells from patients with Wolfram Syndrome	Yes	500 μM 4-PBA and 50 μM TUDCA	Inhibited apoptosis Restored insulin secretion
		Wfs1-KO mice, in vivo	No	Given chow with 4-PBA: 0.338% and TUDCA: 0.225% for 1 month starting from 5 to 6 weeks old	Improved outcome of glucose tolerance test vs. Wfs1-KO mice not given PBA + TUDCA
Meng et al. [30]	EGCG	Cell-free culture with 32 μM hIAPP	Yes	32 μM EGCG	Near-complete elimination of amyloid fibril formation detected by TfT and TEM Disaggregation of existing amyloid fibrils
		Rat INS-1 beta cells cultured with 30 μM hIAPP	Yes	30 μM EGCG	Restoration of cell viability from ~22% to ~77%
Suzuki et al. [29]	EGCG	Cell-free culture with 85 μM hIAPP	Yes	1:5, 1:2, 1:1, and 5:1 molar ratios of EGCG to hIAPP	EGCG diverts amyloid to non-fibrillar aggregates in a dose-dependent manner
Franko et al. [28]	EGCG	hIAPP heterozygous and homozygous mice, in vivo	Yes	3 weeks of EGCG 60 mg/kg administered after the onset of diabetes	In heterozygous mice, decreased amyloid fibril intensity No significant improvement in homozygous mice

## Data Availability

No new data were created or analyzed in this study. Data sharing is not applicable to this article.

## Abbreviations

The following abbreviations are used in this manuscript:

IAPP	Islet amyloid polypeptide
hIAPP	Human islet amyloid polypeptide
mIAPP	Mouse islet amyloid polypeptide
rIAPP	Rat islet amyloid polypeptide
UPR	Unfolded protein response
PBA	4-phenylbutyrate
TUDCA	Tauroursodeoxycholic acid
EGCG	Epigallocatechin gallate
T1DM	Type 1 diabetes mellitus
T2DM	Type 2 diabetes mellitus
TXNIP	Thioredoxin-interacting protein
FoxA2	Forkhead box protein A2
NLRP3	NLR family pyrin domain containing 3
ER	Endoplasmic reticulum
INS	Insulin gene
PC1/3	Prohormone convertase 1/3
IL-1β	Interleukin 1 beta
IRE1α	Inositol-requiring enzyme-1 alpha
PERK	Protein kinase RNA-like ER kinase
ATF6	Activating transcription factor-6
ATF6(N)	N-terminal domain of activating transcription factor-6
eIF2α	Alpha subunit of eukaryotic translation initiation factor-2
ERAD	ER-associated degradation
BiP	Binding immunoglobulin protein
GRP78	78 kDa glucose-regulated protein
JNK	C-jun amino terminal kinase
ATF4	Activating transcription factor-4
CHOP	C/EBP homologous protein
XBP1	X-box binding protein 1
XBP1s	Spliced X-box binding protein 1
UCH-L1	Ubiquitin carboxyl-terminal hydrolase L1
p62	Sequestome 1
Nf-κB	Nuclear factor kappa B
TNF-α	Tumor necrosis factor alpha
BMDC	Bone marrow-derived dendritic cells
miR-17	MicroRNA-17
MAFA	MAF bZIP Transcription Factor A
ChoRE	Carbohydrate response element
ChREBP	Carbohydrate response element binding protein
HIP	Hybrid insulin peptides
TGR5	Takeda G-protein coupled receptor 5
FXR	Farnesoid X receptor
STZ	Streptozocin
HDAC	Histone deacetylase
iPS	Induced pluripotent stem cell
ThT	Thioflavin T
